# Evolution of the NET (NocA, Nlz, Elbow, TLP-1) protein family in metazoans: insights from expression data and phylogenetic analysis

**DOI:** 10.1038/srep38383

**Published:** 2016-12-08

**Authors:** Filipe Pereira, Sara Duarte-Pereira, Raquel M. Silva, Luís Teixeira da Costa, Isabel Pereira-Castro

**Affiliations:** 1 Centro Interdisciplinar de Investigação Marinha e Ambiental (CIIMAR), Universidade do Porto, Porto, Portugal; 2 Instituto de Patologia e Imunologia Molecular da Universidade do Porto (IPATIMUP), Porto, Portugal; 3 Instituto de Ciências Agrárias e Ambientais Mediterrânicas (ICAAM), Universidade de Évora, Évora, Portugal; 4 Instituto de Investigação e Inovação em Saúde (i3S), Universidade do Porto, Porto, Portugal

## Abstract

The NET (for NocA, Nlz, Elbow, TLP-1) protein family is a group of conserved zinc finger proteins linked to embryonic development and recently associated with breast cancer. The members of this family act as transcriptional repressors interacting with both class I histone deacetylases and Groucho/TLE co-repressors. In *Drosophila,* the NET family members Elbow and NocA are vital for the development of tracheae, eyes, wings and legs, whereas in vertebrates ZNF703 and ZNF503 are important for the development of the nervous system, eyes and limbs. Despite the relevance of this protein family in embryogenesis and cancer, many aspects of its origin and evolution remain unknown. Here, we show that NET family members are present and expressed in multiple metazoan lineages, from cnidarians to vertebrates. We identified several protein domains conserved in all metazoan species or in specific taxonomic groups. Our phylogenetic analysis suggests that the NET family emerged in the last common ancestor of cnidarians and bilaterians and that several rounds of independent events of gene duplication occurred throughout evolution. Overall, we provide novel data on the expression and evolutionary history of the NET family that can be relevant to understanding its biological role in both normal conditions and disease.

The NET protein family is a group of conserved zinc finger proteins linked to embryonic development (reviewed in ref. 1) and more recently to cancer^2^,^3^,^4^. The term NET derives from the names of the first proteins discovered in this family: NocA, Nlz, Elbow, and TLP-1^1^. *NocA* (no ocelli) and *Elbow* are paralogous genes located 82 kb apart on chromosome 2L of *Drosophila melanogaster*^5^. The NocA and Elbow proteins share 35% of sequence identity and are both implicated in retina, trachea, wing and leg development. NocA is also important for the development of the *Drosophila*’s embryonic brain and ocellar structures^6^,^7^,^8^,^9^. Only one NET member (TLP-1; T lineage defect, LePtoderan tail) is found in *Caenorhabditis elegans*, and it is involved in asymmetric cell fate determination and morphogenesis during the development of the nematode tail^10^. Vertebrates have two NET family members, the ZNF703 (also known as Nlz1) and ZNF503 (also known as Nlz2) paralogous proteins, which were first described in zebrafish^11^,^12^. Studies on zebrafish, chicken and mouse have demonstrated that these proteins are widely expressed during embryogenesis in the brain, spinal cord, face, limbs and somites and participate in developmental processes that include optic fissure closure during eye development^13^, limb formation^14^, motoneuron identity specification^15^, hindbrain patterning^11^,^12^,^16^,^17^,^18^,^19^ and striatum development^16^,^20^. Although the expression of the two NET members during embryonic development in humans remain uncharacterized, we have previously shown that *ZNF703* is expressed ubiquitously in human adult tissues^21^. The NET family has gained further relevance by the discovery that *ZNF703* is a luminal B human breast cancer oncogene^2^,^3^, with the 8p11-12 chromosomal region where *ZNF703* is located being frequently amplified in breast cancer cases^22^,^23^,^24^,^25^. Furthermore, overexpression of the mouse *Znf703* is associated with breast cancer progression and metastasis^26^. There is also evidence that *ZNF703* acts as an oncogene that promotes progression in gastric cancer^4^.

Several studies have shown that NET proteins function as transcription repressors^15^,^18^,^19^,^21^,^26^ and are unlikely to directly bind DNA^1^. Moreover, the NET proteins interact with known players of the repression process in different species, such as Groucho family corepressors^7^,^12^,^15^,^19^,^21^,^26^ and class I histone deacetylases HDAC1 and HDAC2^12^,^18^. It was recently shown that ZNF703 can repress TGFBR2 (transforming growth factor β receptor II) and E-cadherin expression^2^,^26^ as well as human TGF-β and TCF/β-catenin-mediated transcription^21^,^26^. Our previous work suggests that vertebrate NET proteins have conserved domains that are important for their function as repressors and nuclear localization^21^.

Despite the role of the NET protein family in critical developmental processes and its association with human breast oncogenesis, the evolutionary history of this family remains poorly understood. Here, we sought to determine the expression of NET members in the diversity of metazoan lineages, refine the NET protein-conserved domains and reconstruct the phylogeny and gene arrangement around NET family genes in metazoans.

## Results

### The NET family is expressed in diverse metazoan lineages

The search for proteins belonging to the NET family in different databases (Ensembl, NCBI and JGI) allowed us to recover 165 protein sequences from different metazoan lineages ranging from cnidarians to vertebrates (Supplementary Table S1). We were unable to identify any NET protein sequence in non-metazoan groups like choanoflagellates (*Monosiga brevicollis* and *Salpingoeca rosetta*) and in metazoan organisms belonging to Placozoa (*Trichoplax adhaerens*), Porifera (*Amphimedon queenslandica*) and Ctenophora (*Mnemiopsis leidyi* and *Pleurobrachia bachei*). Although gaps in the genomic sequences may explain the absence of these proteins in databases, it is likely that the genes encoding NET proteins might have appeared only in the common ancestor of Eumetazoa.

We identified NET proteins in various invertebrate groups where this family has never been documented before (Supplementary Table S1), including Cnidaria and Brachiopoda with one NET protein; Annelida with two NET proteins and Mollusca with one (*Octopus bimaculoides* and *Aplysia californica*), two (*Crassostrea gigas*) or three NET proteins (*Lottia gigantea*). The deuterostomes *Saccoglossus kowalevskii, Ptychodera flava* (Hemichordata) and *Strongylocentrotus purpuratus* (Echinodermata) have one NET protein each (Supplementary Table S1). In chordates, a single NET protein was found in Urochordata (*Ciona intestinalis*) and Cephalochordata (*Branchiostoma floridae*). In Vertebrata two NET family proteins (the paralogous proteins ZNF703 and ZNF503; Supplementary Table S1) are present in all species, with the exception of Cyclostomata (*Lethenteron japonicum*) with a single NET protein. We have also observed that several fish species have two Znf703 (Nlz1) and two Znf503 (Nlz2) proteins (Supplementary Table S1), most likely due to the fish-specific whole genome duplication (WGD) event^27^.

**Figure 1:**
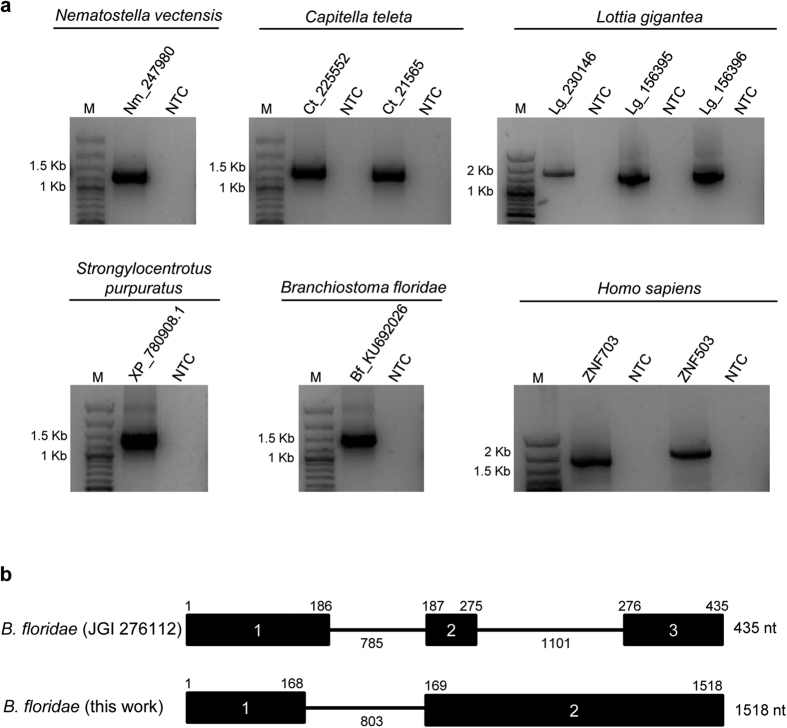
Expression of NET family genes in different metazoan species. (**a**) RT-PCR analysis of transcripts from the NET family shows broad expression in different metazoan groups. Human *ZNF703* was used as positive control. NTC: RT-PCR non-template control; M: molecular-weight size marker. (**b**) Schematic illustration of the *B. floridae* gene structure as deduced by sequencing of the PCR product depicted in (**a**) and of the transcript deposited in the JGI genome portal (accession number 276112). The sequence length is shown for the exons above the black rectangles and for the introns below the thin line. The total transcript length is shown at the right of each scheme. Nt: nucleotide.

RT-PCR and sequencing analyses were used to determine whether NET family members are expressed in metazoan groups where they had not been studied previously. As shown in Fig. 1a, NET family genes were found to be expressed in *Nematostella vectensis* (starlet sea anemone), *Capitella teleta* (polychaete worm), *L. gigantea* (owl limpet), *S. purpuratus* (purple sea urchin) and *B. floridae* (amphioxus) (Fig. 1a). We were able to show that *L. gigantea* has indeed three NET proteins, as suggested by the inspection of its genome (Fig. 1a and Supplementary Table S1). In addition, we also demonstrate here that *ZNF503* is expressed in humans (Fig. 1), as previously shown for *ZNF703* where the gene is ubiquitously expressed in adult human tissues and in cancer cell lines^21^.

The results indicate that the amphioxus transcript has a 1518 nucleotide-long coding sequence with several differences in relation to the transcript from the *B. floridae* genome assembly v.1.0 available at the JGI genome portal (Fig. 1a and b). The sequence determined by us (GenBank accession number KU692026) shows that exon 1 is shorter than indicated at the JGI portal due to the use of a donor splice site (GT) located 18 nucleotides upstream of the donor splice site indicated in the JGI sequence (Fig. 1b). Moreover, the transcript from our sample implies that the gene has only two exons, with exon 2 having 1350 nucleotides that are separated in intron 2 and exon 3 in the JGI transcript (Fig. 1b). The large exon 2 of our sample includes the nucleotides that code for the important C_2_H_2_ zinc finger domain characteristic of all proteins belonging to the NET family^1^,^21^, which are located in what is annotated as intron 2 of the JGI portal. Our results suggest that the amphioxus coding sequence encodes a NET protein with 506 amino acids instead of the 144 amino acids reported in the JGI portal. The JGI transcript might be an alternative splicing transcript, possibly encoding a non-functional protein, or result from an erroneous automatic annotation.

Overall, these results demonstrate that NET family members originated in the last common ancestor to Eumetazoa and are present in the genomes of the majority of metazoan lineages.

### NET family gene organization and protein domains

The comparison of 31 different NET family genes shows that they are typically composed by two exons (Supplementary Table S2), with exon 2 usually larger than exon 1 (Fig. 1b depicts an example of such gene organization). Only four cases (13%) among the 31 different species lack the two exon structure, namely *tlp-1 (C. elegans)* with 4 exons and *Elbow (D. melanogaster), ZNF703 (Monodelphis domestica)* and *Znf703 (Mus musculus)* with 3 exons. This result suggests that the ancestral gene from which all others derived most likely comprised 2 exons. Close inspection of the intron(s) reveals that NET genes have a conserved intron position and phase (Supplementary Table S2). Usually, NET genes have a single phase 0 intron located 5 to 6 codons upstream the nucleotides that code for the Sp protein domain. The location of the phase 0 intron is also maintained in species with more than one intron. Despite the high sequence variability evident in the phase 0 intron, the exon sequences flanking the intron are conserved (Supplementary Figure 1). The post-intron codons are conserved across all taxonomic groups (Supplementary Figure 1), while the pre-intron codons are conserved within some taxonomic groups (e.g., the VSPIE amino acid sequence is shared by tetrapods and the PLPTT sequence occurs in most annelids, arthropods and mollusks).

**Figure 2:**
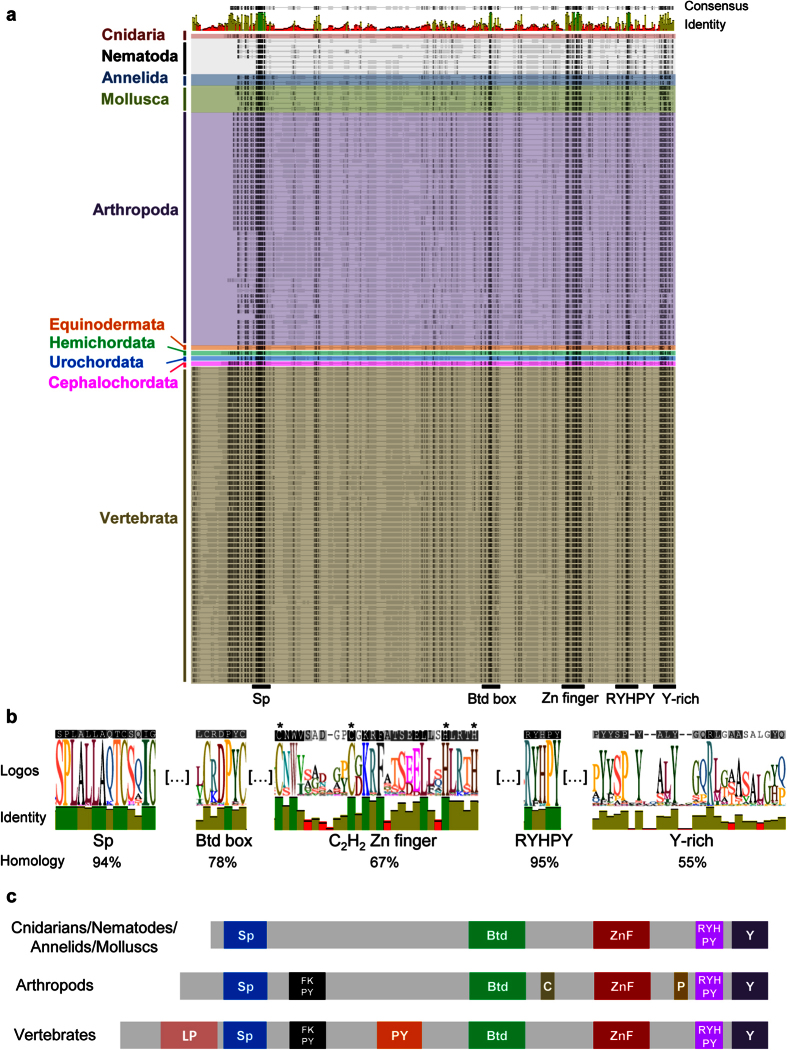
NET family protein domains. (**a**) General overview of the protein sequence alignment using metazoan NET proteins. The names and locations of the five domains are shown below the alignment. Species were grouped according to their taxonomic group using different colours. Taxon name is shown to the left of each group. The consensus sequence and identity is indicated for every position above the alignment, with high and low identity values represented by green and red bars, respectively. (**b**) Amino acid sequences of the five conserved domains identified in the alignment in (**a**). A high degree of conservation is evident in the sequence logos, sequence identity and percentage of pairwise identity (homology). The consensus sequence of each domain is shown above the logos. The two cysteines and two histidines characteristic of the C_2_H_2_ zinc finger domain are highlighted by an asterisk. (**c**) Schematic representation of the domains present in the different taxonomic groups. Btd – Buttonhead box; C – cysteine-rich domain; ZnF – C_2_H_2_ zinc finger domain; P – proline-rich domain and Y – tyrosine-rich domain.

The alignment of NET proteins allowed the identification of five highly conserved protein regions with a high percentage of pairwise identity: the Sp, Btd box, C_2_H_2_ Zinc finger, RYHPY and Y-rich (tyrosine-rich) domains (Fig. 2a and b). These five regions were found in all NET proteins and can be defined as the NET family core protein domains.

The Sp, Btd box and C_2_H_2_ Zinc finger domains were previously described and are common to both NET and Sp protein families^1^. The RYHPY and Y-rich domains result from the split of the formerly designated C-terminal YL domain, described by us in vertebrate NET proteins^21^ due to the presence of a highly variable region between them. These two domains are not shared with the Sp protein family. By in-depth analysis of the protein sequence alignment we found that the Sp domain can be defined by a stretch of 14 amino acids with the consensus sequence S-P-L-[A/E]-[L/M]-L-A-[Q/A/K]-T-C-[S/E/N]-X-I-G (Fig. 2b). Furthermore, our data suggests that the previously defined Btd box consensus sequence R-X_0–4_-C-X-[C/D/N]-P-[N/Y]-C is not conserved in all NET family members, being more divergent than in the Sp family or in the Btd protein from *D. melanogaster,* where it was originally found^1^,^28^,^29^. In fact, the R-X_0–4_ residues at the initial portion of the domain were only present in nematodes and cnidarians. We were able to redesign the consensus sequence of the Btd box domain to a 7-amino-acid stretch with 78% pairwise identity: X-[C/S]-X-[D/N/E]-P-X_1-2_-C (Fig. 2b). All species have a cysteine (C) in the second position of the consensus sequence with the exception of *C. intestinalis*, which has a serine (S) in that position.

The NET protein family differs from the Sp family by having only one zinc finger from the C_2_H_2_ type, making them unlikely to directly bind DNA^1^. Our analyses suggests that the zinc finger domain differs from the usual C_2_H_2_ zinc finger consensus sequence F/Y-X-C-X_2–5_-C-X_3_-F/Y-X_5_-ϕ-X_2_-H-X_3–5_-H, where Φ indicates a hydrophobic residue and X any amino acid^1^, by having more residues (eight or nine) between the two cysteines along with some residues highly conserved between the second cysteine and the first histidine. Therefore, the consensus sequence of the C_2_H_2_ zinc finger domain of the NET family can be more accurately represented as X_2_-**C**-[N/S]-W-X_6-7_-**C**-[G/D]-K-[R/S/V]-F-X_4_-[E/D]-L-X_2_-**H**-X_3-4_-**H** (Fig. 2b).

Finally, two additional conserved domains at the C-terminal portion of the NET proteins were identified, that we named RYHPY and Y-rich domain according to the most abundant amino acids present in those domains (Fig. 2b). The RYHPY domain has the consensus sequence R-[Y/F]-[H/N/R]-P-Y, and the Y-rich domain has four tyrosines conserved in almost every species (Fig. 2b).

Although all NET proteins possess the five core domains (Fig. 2a and b), our analyses suggest the existence of specific domains in certain taxonomic groups, as already described for the vertebrate LP and PY domains^21^. Indeed, the alignment of proteins from arthropods allowed the discovery of two lineage-specific domains in this group: a cysteine-rich (C-rich) domain located upstream of the Btd box and a proline-rich (P-rich) domain located downstream of the zinc finger domain (Fig. 2c). Both arthropods and vertebrates share a FKPY motif placed after the Sp domain that is 100% conserved (Fig. 2c). This motif was previously named ‘Groucho-binding domain’, but recent data indicates that is not the domain required for the interaction between NET and Groucho/TLE proteins, at least not in vertebrates^15^,^18^,^19^,^21^. Finally, the previously described LP and PY domains^21^ were only found in vertebrates (Fig. 2c).

In summary, we identified five core protein domains with new consensus sequences by using the largest protein sequence alignment ever performed with metazoan NET proteins. The lineage-specific domains identified here should also be instrumental for functional studies of NET proteins in these specific taxonomic groups.

### The phylogeny of the NET protein family

**Figure 3:**
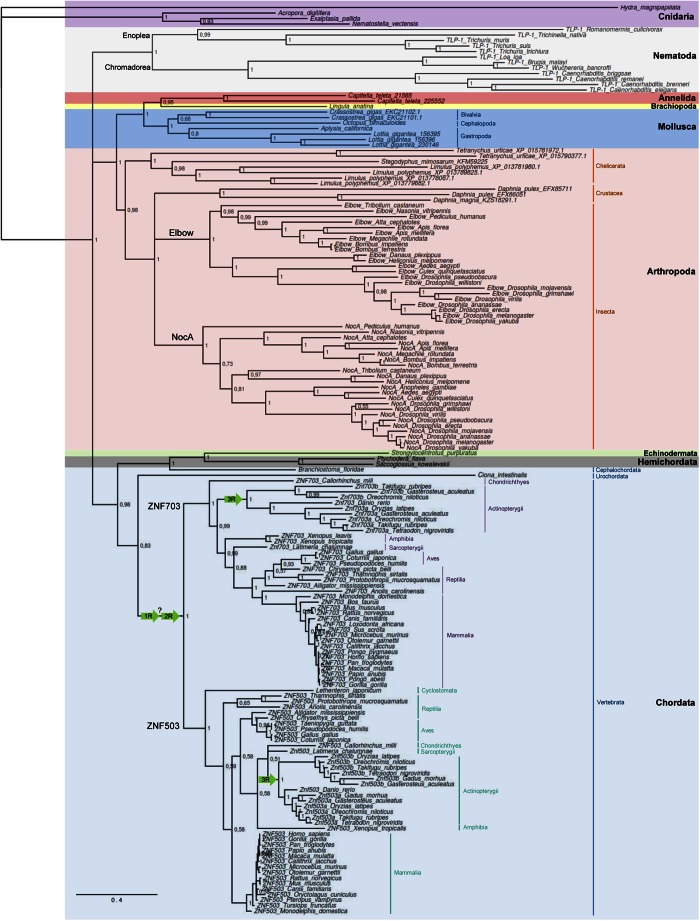
Bayesian phylogenetic tree built with metazoan NET protein sequences (n = 165). Bayesian posterior probabilities are shown on the nodes. The scale bar indicates substitutions per site. Arrows indicate where whole genome duplications (WGDs) might have occurred.

The inferred phylogenetic tree built with NET protein sequences has cnidarians as an outgroup, with *Hydra magnipapillata* forming a separate branch from the other cnidarian species (Fig. 3). The nematodes form a monophyletic group, with a clear separation between Enoplea (*Romanomermis culicivorax, Trichinella native, Trichuris* sp.) and Chromadorea (*Brugia malayi, Caenorhabditis sp., Loa loa* and *Wuchereria bancrofti*), supported by a Bayesian posterior probability (PP) of 1. The NET proteins from Annelida, Brachiopoda, Mollusca and Arthropoda form separate clades, supported by PP higher than 0.95. Two NET paralogues were found in *C. teleta* (Annelida), clustering with the brachiopod *Lingula anatina*. The sister group to clade Annelida/Brachiopoda is Mollusca (PP = 1), which forms a monophyletic group that includes species from Bivalvia, Cephalopoda and Gastropoda. We found mollusc species with one (*O. bimaculoides* and *A. californica*), two (*C. gigas*) or three (*L. gigantea*) NET paralogues (Fig. 3).

**Figure 4:**
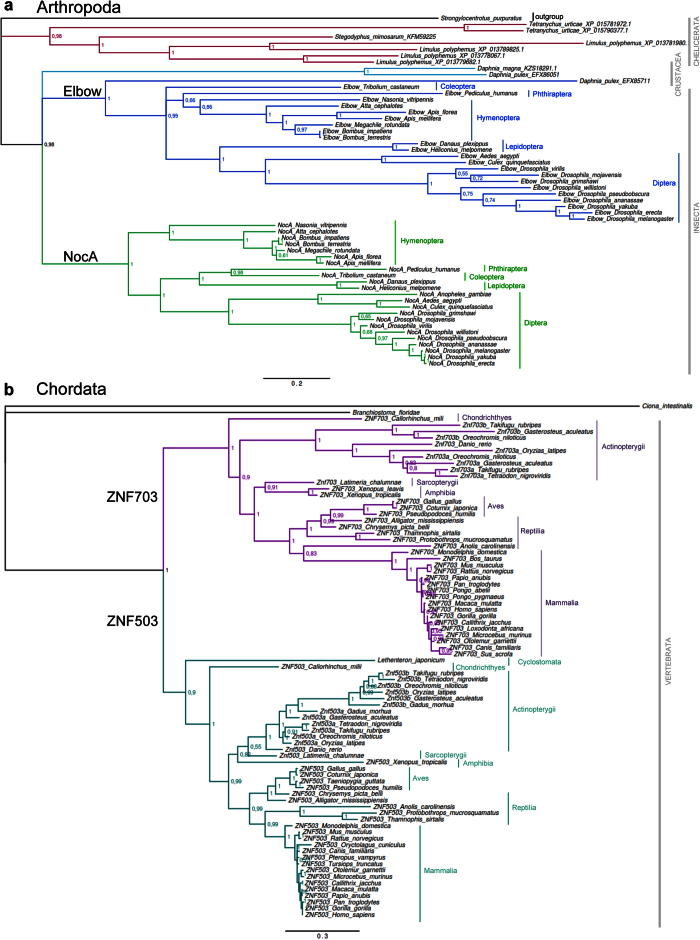
Bayesian phylogenetic tree built with (**a**) Arthropod and (**b**) Chordate NET protein sequences. Bayesian posterior probabilities are shown on the nodes. The scale bar indicates substitutions per site.

The phylogenetic trees built with all NET protein sequences (Fig. 3) and exclusively with arthropods (Fig. 4a) include all chelicerates as a monophyletic group (PP of 0.98 for the split from the Crustacea/Insecta clade). We found two NET paralogues in *Tetranychus urticae* and four in *Limulus polyphemus*. Two NET paralogues were found in *Daphnia pulex*, one of them clustering with the single sequence found in *Daphnia magna*. The other *D. pulex* protein either clusters with Elbow sequences from insects (Fig. 4a) or forms a separate branch (Fig. 3). The trees clearly show that the paralogues Elbow and NocA form two well-supported distinct clades (PP of ~1). The NET proteins from species of the same order cluster together inside each paralogue, with most bifurcations supported by high posterior probabilities. The only species with a single NET protein in this group was *Anopheles gambiae*, with the only identified protein clustering with NocA. The lack of the Elbow paralogue in this mosquito may be due to gene loss or to an incomplete genomic sequence or assembly error as all other insects possessed the two paralogues.

The NET proteins from deuterostomes form a single clade separated from protostomes, supported by a PP of 1 (Fig. 3). The representative species of Hemichordata (*S. kowalevskii* and *P. flava*) and Echinodermata (*S. purpuratus*) cluster together (PP of 1), supporting the existence of a supraphyletic clade named Ambulacraria^30^. The NET protein of the Cephalochordata *B. floridae* clusters with Hemichordata and Echinodermata (PP of 1), while the protein from the Urochordata representative (*C. intestinalis*) clusters with vertebrates. The phylogenetic trees built exclusively with chordates (Fig. 4b) and with all NET proteins (Fig. 3) show a clear separation between the ZNF703 and ZNF503 paralogues with a PP of 1. In the ZNF703 branch, the first bifurcation separates the NET protein of the ghost shark *Callorhinchus milii* (Chondrichthyes) from the remaining species (PP = 1). The NET proteins from Actinopterygii are organized in a monophyletic group. The representative of Sarcopterygii, the coelacanth *Latimeria chalumnae,* is positioned close to amphibians in both trees. The NET proteins from Aves, Reptilia and Mammalia are arranged in a single branch of the tree. The single NET protein retrieved for Cyclostomata (*L. japonicum*, the Japanese lamprey) clusters with ZNF503 proteins in both trees (PP of 0.9 and 1). The absence of a ZNF703 paralogue may indicate that the Japanese lamprey has only a single NET protein as observed in Urochordata and Cephalochordata. However, the clustering of the single NET protein with ZNF503 suggests that there might have been a loss of the ZNF703 paralogue in *L. japonicum*, or that there is an incomplete genome sequence.

The phylogenetic relationship between ZNF503 and ZNF703 proteins is similar in the tree built exclusively with chordates (Fig. 4b). However, several differences were observed between ZNF503 and ZNF703 in the tree will all NET proteins (Fig. 3), with most nodes having a weak statistical support (PP lower than 0.60). Overall, inside each paralogue clade, the NET proteins of species from the same class cluster together with strong statistical support.

### The independent duplication events of NET genes

The phylogenetic analyses confirmed the occurrence of multiple independent gene duplication events in the NET family (Figs 3 and 4). The gene duplications in species of Annelida (*C. teleta*), Mollusca (*C. gigas* and *L. gigantea*) and Arthropoda (*T. urticae* and *L. polyphemus*) resulted in paralogues that cluster together, separated from orthologues. Interestingly, the duplications observed in *L. gigantea* occurred in the same genomic region, as the three paralogues are located close to each other in the SuperContig LOTGIsca_11 (Supplementary Fig. S2). This gene cluster was most likely created by two rounds of tandem gene duplication events, including an inversion that generated the *L. gigantea_156396* and *L. gigantea_230146* genes that cluster together in the phylogenetic tree (Fig. 3) and are neighbours with different orientations in their genomic locus (Supplementary Fig. S1). Tandem duplications were also at the origin of the two NET paralogues present in *C. teleta, C. gigas and D. pulex,* as shown in Supplementary Fig. S2.

The duplication detected in Arthropoda generating the *Elbow* and *NocA* paralogues form distinct clusters with all species. We found that the *Elbow* and *NocA* paralogues in insects are located in the same chromosomal region in opposite directions. In some species, they are adjacent to each other (e.g. *Culex quinquefasciatus* and *Pediculus humanus*), whereas in others (e.g., *Tribolium castaneum, Nasonia vitripennis* and *D. melanogaster*) additional genes are located between the two paralogues (Supplementary Fig. S2). For example, in *D. melanogaster, Elbow* and *NocA* are 82 Kb apart with several genes between them. This result suggests that relocation of new genes to the region between the two paralogues occurred in some lineages after the ancestral duplication event.

**Figure 5:**
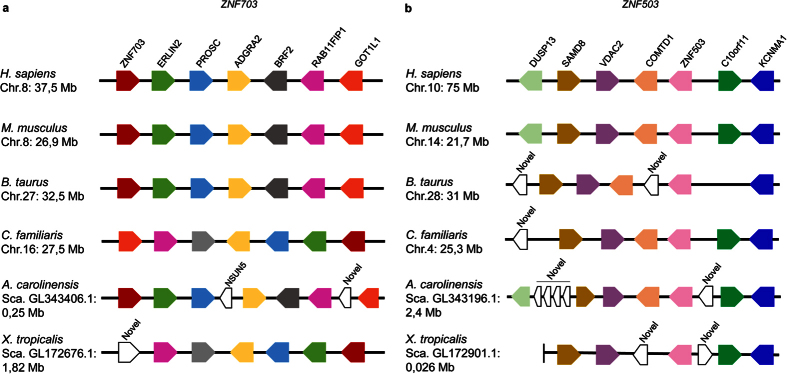
Synteny conservation around *ZNF703* (**a**) and *ZNF503* (**b**) genomic regions in six representative species of tetrapods. The schemes show the relative position and orientation of the NET family and neighbouring genes. The sizes and distances between genes are not to scale. Numbers indicate chromosomal (Chr.) or scaffold (Sca.) locations in megabases (Mb) of the first gene analysed. Colour code represents orthologous relationship among genes. In (**b**) the scaffold of *X. tropicalis* terminates after *SAMD8,* which is indicated by a vertical line.

The duplication observed in chordates generating the *ZNF703* and *ZNF503* paralogues also occurred before speciation within this group (Fig. 3). The possible modes of gene duplication in vertebrates were analysed by examining the gene organization surrounding the NET genes. We found synteny in both the *ZNF703* (Fig. 5a) and *ZNF503* (Fig. 5b) genomic regions in the six tetrapod species analysed. Although the degree of syntenic conservation is high in both genomic regions, the *ZNF703* locus has a more conserved arrangement of genes than *ZNF503*. For example, *ERLIN2* and *PROSC* are close to *ZNF703* in all species (Fig. 5a), whereas the gene order in *B. taurus* is similar to humans around *ZNF703* but differs in the *ZNF503* region (Fig. 5a and b). Curiously, the gene order surrounding *ZNF703* in *Canis familiaris* and *Xenopus tropicalis* is the same as in *Homo sapiens* but with genes annotated in the opposite strand (Fig. 5a).

**Figure 6:**
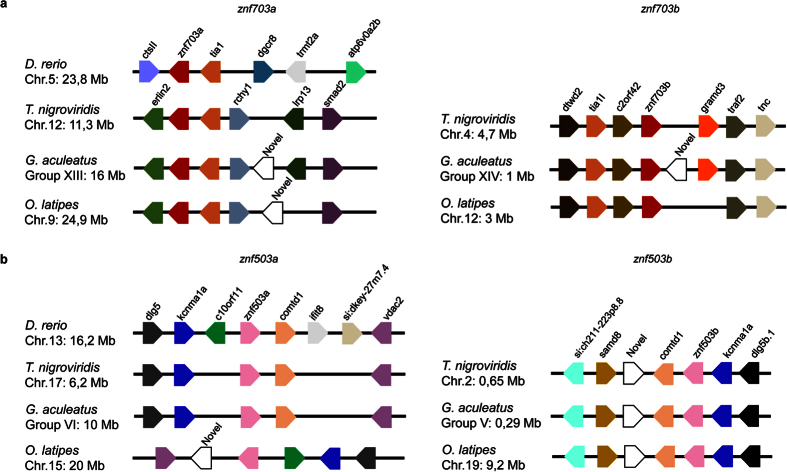
Synteny conservation around *znf703a* and *znf703b* (**a**) or *znf503a* and *znf503b* (**b**) genomic regions in representative species of fish. The schemes show the relative position and orientation of the NET and neighbouring genes. The distances between genes and the sizes are not to scale. Numbers indicate chromosomal (Chr.) or scaffold (Sca.) locations of the first gene analysed in megabases (Mb). Colour code represents orthologous relationship among genes, including with the genes of Fig. 5.

In fish, a conserved synteny was observed between *Tetraodon nigroviridis, Gasterosteus aculeatus* and *Oryzias latipes* in all loci analysed (Fig. 6). The synteny around *znf703a* and *znf503a* loci was less evident in *Danio rerio* (Fig. 6), particularly for the *znf703a* locus. We did not find *znf703b* and *znf503b* genes in *D. rerio* (Fig. 6), suggesting that it might have lost these genes after the fish-specific WGD event. Overall, the conserved synteny around the *znf703a* and *znf703b* (Fig. 6a) as well as *znf503*a and *znf503b* (Fig. 6b) genomic regions supports the 3R genome duplication as the origin of fish NET paralogues.

## Discussion

Here, we presented the first comprehensive study of the evolutionary pattern of the NET protein family in metazoans by using expression analysis, comparative genomics and phylogenetic inferences. The expression of NET family members in species representing the majority of the metazoan groups suggests that the NET family emerged with the formation of cnidarians and bilaterians and that it plays an important functional role throughout Eumetazoa evolution. Accordingly, NET family members are known to repress Wnt and TGF-β mediated transcription^21^,^26^,^31^, which are important signalling pathways expressed in all major extant metazoan lineages^32^,^33^,^34^. Moreover, NET members interact with Groucho/TLE co-repressors^7^,^12^,^15^,^18^,^19^,^21^,^26^, which are found in all metazoan organisms^35^. These multiple interactions suggest that these families could co-operate in the embryonic development of most animals, explaining the conservation of the NET family across different taxonomic groups. The NET family members are also conserved in terms of gene structure, with most of them having two exons with a phase 0 intron in-between the coding sequence, which keeps codons intact. In addition, the codons that flank the phase 0 intron have a high degree of sequence conservation. This gene organization was most likely the structure of the NET ancestral gene. The conservation across different taxonomic groups suggests that NET genes may be under constraint to maintain this arrangement.

Our analyses also recognized conserved regions in NET proteins across species. We identified 5 core protein domains present in the NET proteins of all metazoan species analysed (Sp, Btd box, C_2_H_2_ Zinc finger, RYHPY and Y-rich domains), which might be relevant elements of the protein’s structure and/or important regions for protein-protein interactions, subcellular localization or other important cellular processes. Although the functions of these protein domains in NET family members are not clearly understood, some studies have suggested that the Sp domain might have a role on transcriptional repression^36^ and the deletion of N-terminal sequences including the Sp domain leads to dominant negative Elbow proteins^9^. The function of the Btd box remains unknown, but it may be required for transcriptional activation^28^. The single C_2_H_2_ Zinc finger present in NET proteins is unlikely to make the NET proteins capable of binding DNA directly. It may instead mediate protein-protein interactions as described for other zinc fingers^37^. The zinc finger of *Drosophila*’s Elbow protein is crucial for its function because mutations in this domain transform the protein into a dominant-negative form^9^. The RYHPY and Y-rich domains are located in the C-terminal region of NET proteins, which is required for the nuclear localization of these transcriptional repressors^12^,^15^,^19^,^21^,^26^,^38^. Given that NET proteins lack a classical NLS^21^, the amino acid motifs present in the RYHPY and/or Y-rich domains could be responsible for NET localization. Moreover, we identified lineage-specific domains that might represent specific protein functions in some taxonomic groups. For example, the PY domain present in vertebrates is important for nuclear localization, though not essential^21^, while the FKPY motif seems to be relevant for binding to Groucho in insects^7^ but not in vertebrates^15^,^19^,^21^.

The phylogenetic relationships among NET family members showed that the two groups of paralogues (Elbow and NocA in Arthropoda and ZNF703 and ZNF503 in Chordata) form two similar well-supported distinct clades. We discovered that the NET family was expanded by independent gene duplications, which are important sources of genomic novelty and complexity^39^. Our analyses revealed that most species possess at least two different NET proteins. The gene duplications observed in Annelida (*C. teleta*), Mollusca (*C. gigas* and *L. gigantea*) and Arthropoda (*T. urticae and L. polyphemus*) were independent because the paralogues in each species are more similar between them than with the copies of other species. If a single duplication had occurred prior to the separation of these taxonomic groups, one would expect two gene clusters each-one with all species and one for each gene copy. However, the clustering of duplicated genes in each species suggests their origin after speciation. The most likely molecular mechanism leading to new duplicates in these taxonomic groups was unequal crossing-over during homologous recombination generating tandem or closely located gene duplicates.

The duplication detected in arthropods generating the *Elbow* and *NocA* paralogues and in chordates generating the *ZNF703* and *ZNF503* paralogues occurred before speciation inside each group because the paralogues form distinct clusters with all species. In vertebrates, the molecular mechanism leading to the *ZNF703* and *ZNF503* duplicates was probably two rounds of WGD events known as 1 R and 2 R that occurred early during vertebrate evolution^39^,^40^,^41^. It was shown that the paralogons containing the *ZNF703* and *ZNF503* paralogues are the result of *en bloc* duplications that occurred after the protostomian-deuterostomian divergence and before the osteichthyian split^42^. In addition, the conserved synteny observed in the genomic regions surrounding each paralogue further supports the WGD hypothesis as the mechanism behind the origin of NET vertebrate paralogues. The duplications detected in the NET paralogues of fish species generating the *Znf703a, Znf703b, Znf503a* and *Znf503b* paralogues are most likely related to the fish-specific WGD event that occurred in the teleost lineage (3R), estimated to have occurred 226–350 million years ago^41^.

Overall, the results presented in this study will significantly contribute to understanding the regulatory and functional plasticity of NET proteins in metazoan evolution.

## Methods

### Identification of NET family protein sequences

A total of 165 complete NET protein sequences belonging to nine metazoan phyla were retrieved from the Ensembl (www.ensembl.org), NCBI (www.ncbi.nlm.nih.gov) and Joint Genome Institute (JGI) (http://genome.jgi.doe.gov/) databases. Sequences from different taxonomic groups were used as queries in TBLASTN or BLASTP searches against the NCBI (non-redundant protein sequences) and JGI (model proteins or filtered model proteins) databases to collect the maximum number of proteins. In the Ensembl database, all of the suggested orthologues of the human ZNF703 and ZNF503 proteins were retrieved. In order to have accurate multiple sequence alignments and reliable phylogenetic inferences, only complete protein sequences were further considered for the analyses. The proteins’ names, accession numbers and source organism (species) are listed in Supplementary Table S1. Proteins with no established designation were named with the species name or with the species name plus the protein accession number when more than one NET protein was present in the same species. Although the NET proteins in fish were previously named Nlz1 and Nlz2, here we used the designations Znf703 and Znf503 to reflect the nomenclature currently in use in all vertebrate species. Suffixes “a” and “b” were used to differentiate the duplicated Znf703 and Znf503 proteins found in some fish species.

### Expression analysis

Samples from *N. vectensis* (specimen S13115, whole organism), *C. teleta* (specimen S13061, whole organism), *L. gigantea* (specimen S13017, foot and mantle), *S. purpuratus* (specimen S13034, gonad) and *B. floridae* (specimen S13045, whole organism) were obtained from the Ocean Genome Legacy (OGL) Database, The Ocean Genome Legacy Center of New England Biolabs, Northeastern University, U.S.A., published on the web at: http://www.northeastern.edu/marinescience/ogl/catalog/. Total RNA was extracted using the Illustra triplePrep kit (GE Healthcare) according to the manufacturer’s instructions. Total RNA from a human spinal cord tissue was obtained from the Human Total RNA Master Panel II (Clontech). To remove genomic DNA contamination from the RNA, 1 μg of total RNA was digested using 1U of DNase I (Fermentas) at 37 °C for 30 min followed by inactivation of the enzyme at 65 °C for 10 min in the presence of EDTA according to the manufacturer’s procedure.

Complementary DNA (cDNA) was synthesized from 1 μg of RNA using the RETROscript Reverse Transcription Kit (Ambion) with oligo(dT) primers (50 μM) according to the manufacturer’s instructions. Reverse-transcription PCR (RT-PCR) assays were prepared using 12.5 μL of HotStarTaq Master Mix Kit (Qiagen), 6% of DMSO (Fermentas), 0.4 μM of each NET species-specific primer (Supplementary Table S3), 7.5 μL of RNase-free water and 1.5 μL of cDNA in a final reaction volume of 25 μL. The amplification conditions comprised a touchdown PCR with an initial denaturation step of 15 min at 95 °C followed by 3 cycles of 30 s at 95 °C, 45 s at T1 and 2 min at 72 °C; 3 cycles of 30 s at 95 °C, 45 s at T2 and 2 min at 72 °C; 33 cycles of 30 s at 95 °C, 45 s at T3 and 3 min at 72 °C, and a final extension step of 10 min at 72 °C in a MyCycler thermocycler (Bio-Rad Laboratories). The T1-T2-T3 annealing temperatures for each primer pair are listed in Supplementary Table S3. The amplification products were visualized on 1% agarose gels, and the image acquisition was processed with Quantity-One 1-D Analysis Software Version 4.6.8 (Bio-Rad Laboratories). The RT-PCR products were purified with ExoSAP-IT (USB Corporation) by incubation at 37 °C for 15 min followed by enzyme inactivation at 85 °C for 15 min. The resulting purified fragments were sequenced in a ABI Prism 3130XL Sequencer (Applied Biosystems) using NET species-specific primers (Supplementary Table S3) and a protocol previously described^43^.

### Sequence alignments and phylogenetic analyses

Three multiple-sequence alignments were performed using the default settings of the MUSCLE 3.6 software^44^ implemented in Geneious v5.5 (http://www.geneious.com)^45^: (1) all NET proteins sequences (*n* = 165); (2) arthropod NET proteins (*n = *55) plus a sequence of *S. purpuratus* (Echinodermata) as an outgroup and (3) chordate NET proteins (*n* = 81). The best amino acid substitution models for the phylogenetic analyses were estimated from the alignments using ProtTest 3.4.2 software^46^ with a gamma distribution with four rate categories. The VT+I+G+F model was selected to build the phylogenetic trees using the alignments with all sequences and with arthropod sequences. The JTT+G+F model was used for the chordate phylogeny. Bayesian analyses were performed with MrBayes v3.2.6 software^47^,^48^ running on the CIPRES Science Gateway^49^. The Metropolis-coupled Markov chain Monte Carlo process was set such that two independent chains ran simultaneously until reaching an average standard deviation of split frequencies of 0.01, suggesting convergence on a stationary distribution. The analyses reached 3,880,000 generations for the tree with all sequences, 1,540,000 for arthropods and 2,710,000 for chordates. A burn-in value of 0.25 was applied. Trees were edited in FigTree v1.4.2 (http://tree.bio.ed.ac.uk/software/figtree/).

### Synteny analysis

Synteny of the genomic regions surrounding *ZNF703* and *ZNF503* genes was determined in human (*Homo sapiens*), mouse (*Mus musculus*), cow (*Bos taurus*), dog (*Canis familiaris*), green anole (*Anolis carolinensis*) and Western clawed frog (*Xenopus tropicalis*) genomes using the CHSminer 1.1 software^50^. The synteny and locations tools of the Ensembl genome browser were used to infer the synteny in zebrafish (*Danio rerio*), tetraodon (*Tetraodon nigroviridis*), stickleback (*Gasterosteus aculeatus*) and medaka (*Oryzias latipes*) genomes.

## Additional Information

**How to cite this article**: Pereira, F. *et al*. Evolution of the NET (NocA, Nlz, Elbow, TLP-1) protein family in metazoans: insights from expression data and phylogenetic analysis. *Sci. Rep.* **6**, 38383; doi: 10.1038/srep38383 (2016).

**Publisher's note:** Springer Nature remains neutral with regard to jurisdictional claims in published maps and institutional affiliations.

## Supplementary Material

Supplementary Information
