# Supplementary material for: Evolution of the NET (NocA, Nlz, Elbow, TLP-1) protein family in metazoans: insights from expression data and phylogenetic analysis
Source: Sci Rep. 2016 Dec 8;6:38383. doi: 10.1038/srep38383 (PMC5144077; doi:10.1038/srep38383)
Supplement: Supplementary Information [file srep38383-s1.pdf]

Supplementary information

Evolution of the NET (NocA, Nlz, Elbow, TLP-1) protein family in metazoans: insights from expression data and phylogenetic analysis

Filipe Pereira[1], Sara Duarte-Pereira[2†], Raquel M. Silva[2†], Luís Teixeira da Costa[3*],

Isabel Pereira-Castro[2,4,#,*]

[1]Centro Interdisciplinar de Investigação Marinha e Ambiental (CIIMAR), Universidade do Porto, Porto, Portugal

[2]Instituto de Patologia e Imunologia Molecular da Universidade do Porto (IPATIMUP), Porto, Portugal

[3]Instituto de Ciências Agrárias e Ambientais Mediterrânicas (ICAAM), Universidade de Évora, Évora, Portugal

[4]Instituto de Investigação e Inovação em Saúde (i3S), Universidade do Porto, Porto, Portugal

[†]Current address: Departamento de Ciências Médicas, iBiMED & IEETA, Universidade de Aveiro, Aveiro, Portugal

[#]Current address: Gene Regulation Group, i3S/IBMC: Instituto de Investigação e Inovação em Saúde/Instituto de Biologia Molecular e Celular, Universidade do Porto, Porto, Portugal.

*Authors for Correspondence: Luís Teixeira da Costa (luisteixeiracosta@gmail.com) and Isabel Pereira-Castro (isabelpereiracastro@gmail.com)

Supplementary Table S1 – List of the NET proteins names, species and accession numbers used in this study.

Protein	Species scientific name	Species common name	Taxonomic group	Accession number	Database
**ZNF703**	*Homo sapiens*	Human	Chordata	NP_079345.1	NCBI
**ZNF703**	*Pongo abelii*	Sumatran orangutan		XP_002819039.1	NCBI
**ZNF703**	*Papio anubis*	Olive baboon		XP_003902690.1	NCBI
**ZNF703**	*Otolemur garnettii*	Small-eared galago		XP_003796349.1	NCBI
**ZNF703**	*Bos taurus*	Cow		NP_001178409.1	NCBI
**ZNF703**	*Canis familiaris*	Dog		ENSCAFP00000009330	Ensembl
**ZNF703**	*Pan troglodytes*	Chimpanzee		XP_519705.3	NCBI
**Znf703a**	*Takifugu rubripes*	Fugu		ENSTRUP00000038621	Ensembl
**Znf703b**	*Takifugu rubripes*	Fugu		ENSTRUP00000014664	Ensembl
**Znf703**	*Latimeria chalumnae*	coelacanth		ENSLACP00000021279	Ensembl
**ZNF703**	*Gorilla gorilla*	Gorilla		ENSGGOP00000011500	Ensembl
**ZNF703**	*Macaca mulatta*	Rhesus monkey		XP_001088637.1	NCBI
**ZNF703**	*Callithrix jacchus*	Marmoset		XP_002807533.1	NCBI
**Znf703a**	*Oryzias latipes*	Medaka fish		ENSORLP00000019477	Ensembl
**Znf703a**	*Oreochromis niloticus*	Nile tilapia		ENSONIP00000020021	Ensembl
**Znf703b**	*Oreochromis niloticus*	Nile tilapia		ENSONIP00000020374	Ensembl
**ZNF703**	*Mus musculus*	Mouse		NP_001094972.1	NCBI
**ZNF703**	*Monodelphis domestica*	Gray short-tailed opossum		ENSMODP00000013642	Ensembl
**ZNF703**	*Loxodonta africana*	African elephant		XP_003412588.1	NCBI
**ZNF703**	*Sus scrofa*	Pig		ENSSSCP00000016774	Ensembl
**ZNF703**	*Pongo pygmaeus*	Bornean orangutan		ENSPPYP00000020747	Ensembl
**ZNF703**	*Rattus norvegicus*	Rat		NP_001102895.1	NCBI
**ZNF703**	*Protobothrops mucrosquamatus*	Taiwan habu		XP_015675243.1	NCBI
**ZNF703**	*Thamnophis sirtalis*	Common garter snake		XP_013921008.1	NCBI

**ZNF703**	*Chrysemys picta bellii*	Western painted turtle		XP_005286569.1	NCBI
**ZNF703**	*Pseudopodoces humilis*	Tibetan ground jay		XP_005530904.1	NCBI
**ZNF703**	*Coturnix japonica*	Japanese quail		XP_015738662.1	NCBI
**ZNF703**	*Gallus gallus*	Chicken		XP_015152915.1	NCBI
**Znf703a**	*Gasterosteus aculeatus*	Three-spined stickleback		ENSGACP00000017559	Ensembl
**Znf703b**	*Gasterosteus aculeatus*	Three-spined stickleback		ENSGACP00000020850	Ensembl
**Znf703a**	*Tetraodon nigroviridis*	Spotted green pufferfish		ENSTNIP00000014377	Ensembl
**Znf703**	*Xenopus tropicalis*	Western clawed frog		NP_001025678.1	NCBI
**Znf703**	*Xenopus laevis*	African clawed frog		NP_001084448.1	NCBI
**Znf703**	*Danio rerio*	Zebrafish		ENSDARP00000051552	Ensembl
**Znf703**	*Callorhinchus milii*	Ghost shark	Chordata	XP_007882836.1	NCBI
**ZNF703**	*Alligator mississippiensis*	American alligator		XP_014458913.1	NCBI
**ZNF703**	*Anolis carolinensis*	Green anole		XP_003226773.2	NCBI
**ZNF503**	*Homo sapiens*	Human		NP_116161.2	NCBI
**ZNF503**	*Anolis carolinensis*	Green anole		ENSACAP00000008322	Ensembl
**ZNF503**	*Pan troglodytes*	Chimpanzee		XP_001149828.1	NCBI
**ZNF503**	*Oryctolagus cuniculus*	Rabbit		ENSOCUP00000000028	Ensembl
**ZNF503**	*Papio anubis*	Olive baboon		XP_003903775	NCBI
**ZNF503**	*Otolemur garnettii*	Small-eared galago		ENSOGAP00000022174	Ensembl
**ZNF503**	*Bos taurus*	Cow		XP_001256480.2	NCBI
**ZNF503**	*Canis familiaris*	Dog		XP_546176.2	NCBI
**ZNF503**	*Tursiops truncatus*	Atlantic bottle-nosed dolphin		ENSTTRP00000013121	Ensembl
**Znf503b**	*Takifugu rubripes*	Fugu		ENSTRUP00000036395	Ensembl
**Znf503a**	*Takifugu rubripes*	Fugu		ENSTRUP00000008283	Ensembl
**ZNF503**	*Gorilla gorilla*	Gorilla		ENSGGOP00000011002	Ensembl
**ZNF503**	*Macaca mulatta*	Rhesus monkey		XP_001095797.1	NCBI

**ZNF503**	*Callithrix jacchus*	Marmoset	Chordata	XP_002807485.1	NCBI
**ZNF503**	*Protobothrops mucrosquamatus*	Taiwan habu		XP_015675944.1	NCBI
**ZNF503**	*Thamnophis sirtalis*	Common garter snake		XP_013929090.1	NCBI
**ZNF503**	*Chrysemys picta bellii*	Western painted turtle		XP_005278820.1	NCBI
**ZNF503**	*Pseudopodoces humilis*	Tibetan ground jay		XP_005520564.1	NCBI
**ZNF503**	*Coturnix japonica*	Japanese quail		XP_015722341.1	NCBI
**ZNF503**	*Gallus gallus*	Chicken		XP_015143780.1	NCBI
**Znf503a**	*Oryzias latipes*	Medaka fish		ENSORLP00000008248	Ensembl
**Znf503b**	*Oryzias latipes*	Medaka fish		ENSORLP00000010871	Ensembl
**ZNF503**	*Pteropus vampyrus*	Large flying fox		ENSPVAP00000007048	Ensembl
**ZNF503**	*Mus musculus*	Mouse		NP_663434	NCBI
**ZNF503**	*Microcebus murinus*	Lesser mouse lemur		ENSMICP00000009922	Ensembl
**ZNF503**	*Monodelphis domestica*	Gray short-tailed opossum		XP_001364694.1	NCBI
**ZNF503**	*Rattus norvegicus*	Rat		NP_001100720	NCBI
**Znf503b**	*Gasterosteus aculeatus*	Three-spined stickleback		ENSGACP00000002911	Ensembl
**Znf503a**	*Gasterosteus aculeatus*	Three-spined stickleback		ENSGACP00000011754	Ensembl
**Znf503a**	*Tetraodon nigroviridis*	Spotted green pufferfish		ENSTNIP00000011471	Ensembl
**Znf503b**	*Tetraodon nigroviridis*	Spotted green pufferfish		ENSTNIP00000016144	Ensembl
**Znf503**	*Xenopus tropicalis*	Western clawed frog		NP_001072698.1	NCBI
**ZNF503**	*Taeniopygia guttata*	Zebra finch		XP_002196287.1	NCBI
**Znf503**	*Danio rerio*	Zebrafish		ENSDARP00000019426	Ensembl
**Znf503a**	*Gadus morhua*	Atlantic cod		ENSGMOP00000013503	Ensembl
**Znf503b**	*Gadus morhua*	Atlantic cod		ENSGMOP00000000235	Ensembl
**Znf503a**	*Oreochromis niloticus*	Nile tilapia		ENSONIP00000023327	Ensembl
**Znf503b**	*Oreochromis niloticus*	Nile tilapia		ENSONIP00000020374	Ensembl
**Znf503**	*Latimeria chalumnae*	Coelacanth		ENSLACP00000020687	Ensembl

**Znf503**	*Callorhinchus milii*	Ghost shark	Chordata	XP_007896701.1	NCBI
**ZNF503**	*Alligator mississippiensis*	American alligator		XP_006260091.2	NCBI
*******	*Lethenteron japonicum*	Japanese lamprey		JL2980	IMBC, A-Star
*******	*Ciona intestinalis*	Transparent sea squirt		XP_002130246	NCBI
*******	*Branchiostoma floridae*	Amphioxus		276112 and KU692026	JGI and GenBank
**Elbow**	*Drosophila melanogaster*	Fruit fly	Arthropoda	FBpp0089383	Ensembl
**NocA**	*Drosophila melanogaster*	Fruit fly		FBpp0080215	Ensembl
**NocA**	*Anopheles gambiae*	African malaria mosquito		AGAP010223-PA	Ensembl
**NocA**	*Nasonia vitripennis*	Parasitic wasp		XP_001606618.1	NCBI
**Elbow**	*Nasonia vitripennis*	Parasitic wasp		XP_001606588.1	NCBI
**NocA**	*Aedes aegypti*	Yellow fever mosquito		XP_001657853.1	NCBI
**Elbow**	*Aedes aegypti*	Yellow fever mosquito		XP_001661395.1	NCBI
**Elbow**	*Apis mellifera*	Honeybee		XP_393412.3	NCBI
**NocA**	*Apis mellifera*	Honeybee		XP_391818.2	NCBI
**NocA**	*Apis florea*	Dwarf honeybee		XP_003695460.1	NCBI
**Elbow**	*Apis florea*	Dwarf honeybee		XP_003692830.1	NCBI
**NocA**	*Atta cephalotes*	Leafcutter ant		ACEP_00012771-PA	Ensembl
**Elbow**	*Atta cephalotes*	Leafcutter ant		ACEP_00012773-PA	Ensembl
**Elbow**	*Bombus impatiens*	Bumblebee		XP_003486600.1	NCBI
**NocA**	*Bombus impatiens*	Bumblebee		XP_003486601.1	NCBI
**NocA**	*Bombus terrestris*	Buff-tailed bumblebee		XP_003402329.1	NCBI
**Elbow**	*Bombus terrestris*	Buff-tailed bumblebee		XP_003402330.1	NCBI
**Elbow**	*Culex quinquefasciats*	Southern house mosquito		XP_001842450.1	NCBI
**NocA**	*Culex quinquefasciatus*	Southern house mosquito		XP_001842449.1	NCBI
**NocA**	*Danaus plexippus*	Monarch butterfly		EHJ69974.1	NCBI
**Elbow**	*Danaus plexippus*	Monarch butterfly		EHJ69973.1	NCBI
**Elbow**	*Megachile rotundata*	Alfalfa leafcutting bee		XP_003700649.1	NCBI

**NocA**	*Megachile rotundata*	Alfalfa leafcutting bee	Arthropoda	XP_003700648.1	NCBI
**NocA**	*Heliconius melpomene*	Postman Butterfly		HMEL012144-PA	Ensembl
**Elbow**	*Heliconius melpomene*	Postman Butterfly		HMEL012148-PA	Ensembl
**Elbow**	*Tribolium castaneum*	Red flour beetle		XP_974945.1	NCBI
**NocA**	*Tribolium castaneum*	Red flour beetle		XP_974922.1	NCBI
**Elbow**	*Pediculus humanus*	Body louse		XP_002424319.1	NCBI
**NocA**	*Pediculus humanus*	Body louse		XP_002424320.1	NCBI
**NocA**	*Drosophila ananassae*	Fruit fly		XP_001961528.1	NCBI
**Elbow**	*Drosophila ananassae*	Fruit fly		XP_001961532.1	NCBI
**Elbow**	*Drosophila erecta*	Fruit fly		XP_001968886.1	NCBI
**NocA**	*Drosophila erecta*	Fruit fly		XP_001968891.1	NCBI
**NocA**	*Drosophila grimshawi*	Fruit fly		XP_001992867.1	NCBI
**Elbow**	*Drosophila grimshawi*	Fruit fly		XP_001992863.1	NCBI
**Elbow**	*Drosophila pseudoobscura*	Fruit fly		XP_001357381.2	NCBI
**NocA**	*Drosophila pseudoobscura*	Fruit fly		XP_001357377.1	NCBI
**NocA**	*Drosophila virilis*	Fruit fly		XP_002057587.1	NCBI
**Elbow**	*Drosophila virilis*	Fruit fly		XP_002057591.1	NCBI
**Elbow**	*Drosophila willistoni*	Fruit fly		XP_002066442.1	NCBI
**NocA**	*Drosophila willistoni*	Fruit fly		XP_002066451.1	NCBI
**NocA**	*Drosophila yakuba*	Fruit fly		XP_002089296.1	NCBI
**Elbow**	*Drosophila yakuba*	Fruit fly		XP_002089292.1	NCBI
**Elbow**	*Drosophila mojavensis*	Fruit fly		XP_002002939.1	NCBI
**NocA**	*Drosophila mojavensis*	Fruit fly		XP_002002933.1	NCBI
*******	*Tetranychus urticae*	Two-spotted spider mite		XP_015781972.1	NCBI
*******	*Tetranychus urticae*	Two-spotted spider mite		XP_015790377.1	NCBI
*******	*Stegodyphus mimosarum*	African social velvet spider		KFM59225	Ensembl
*******	*Limulus polyphemus*	Atlantic horseshoe crab		XP_013778067.1	NCBI

***	*Limulus polyphemus*	Atlantic horseshoe crab	Arthropoda	XP_013779682.1	NCBI
***	*Limulus polyphemus*	Atlantic horseshoe crab		XP_013781980.1	NCBI
***	*Limulus polyphemus*	Atlantic horseshoe crab		XP_013789825.1	NCBI
***	*Daphnia magna*	Water flea		KZS18291.1	NCBI
***	*Daphnia pulex*	Water flea		EFX85711	NCBI
***	*Daphnia pulex*	Water flea		EFX86051	NCBI
***	*Saccoglossus kowalevskii*	Acorn worm	Hemichordata	NP_001161611.1	NCBI
***	*Ptychodera flava*	Hawaiian acorn worm		pfl_40v0_9_20150316_1g11396.t1	NCBI
***	*Strongylocentrotus purpuratus*	Purple sea urchin	Echinodermata	XP_780908.1	NCBI
***	*Lottia gigantea*	Owl limpet	Mollusca	156395	JGI
***	*Lottia gigantea*	Owl limpet		156396	JGI
***	*Lottia gigantea*	Owl limpet		230146	JGI
***	*Crassostrea gigas*	Pacific oyster		EKC21102.1	NCBI
***	*Crassostrea gigas*	Pacific oyster		EKC21101.1	NCBI
***	*Octopus bimaculoides*	Two-spotted octopus		Ocbimv22007340m.p	Ensembl
***	*Aplysia californica*	California sea hare		XP_005089256.1	NCBI
***	*Lingula anatina*	Common oriental lamp shell	Brachiopoda	XP_013387144.1	NCBI
***	*Capitella teleta*	Polychaete worm	Annelida	225552	JGI
***	*Capitella teleta*	Polychaete worm		21565	JGI
***	*Nematostella vectensis*	Starlet sea anemone	Cnidaria	247980	JGI
***	*Acropora digitifera*	Staghorn coral		XP_015770611.1	NCBI
***	*Exaiptasia pallida*	Pale anemone		KXJ23432.1	NCBI
***	*Hydra magnipapillata*	Hydra		XP_002158451.2	NCBI
**TLP-1**	*Caenorhabditis elegans*	Roundworm	Nematoda	T23G4.1	Ensembl
**TLP-1**	*Caenorhabditis remanei*	Roundworm		XP_003093932	NCBI
**TLP-1**	*Caenorhabditis brenneri*	Roundworm		EGT59238	NCBI

**TLP-1**	*Caenorhabditis briggsae*	Roundworm	Nematoda	XP_002647187	NCBI
**TLP-1**	*Brugia malayi*	Filarial nematode worm		XP_001898960	NCBI
**TLP-1**	*Loa loa*	Eye worm		XP_003144436	NCBI
**TLP-1**	*Wuchereria bancrofti*	***		EJW88402.1	NCBI
**TLP-1**	*Romanomermis culicivorax*	Nematode worm		nRc.2.0.1.t45586-RA	WormBase ParaSite
**TLP-1**	*Trichinella nativa*	***		D917_02260	WormBase ParaSite
**TLP-1**	*Trichuris muris*	Mouse whipworm		TMUE_s0050002400	WormBase ParaSite
**TLP-1**	*Trichuris suis*	Pig whipworm		M514_07390	WormBase ParaSite
**TLP-1**	*Trichuris trichiura*	Human whipworm		TTRE_0000334501	WormBase ParaSite

*** No information available

Supplementary Table S2 - List of the NET genes used for gene structure comparisons.

Gene	Species	Taxonomic group	Accession number	Database	Number of exons	Number of introns	Intron(s) location(s)	Intron(s) phase
ZNF703	*Homo sapiens*	Mammalia	NM_025069.1	NCBI	2	1	Within coding region	0
ZNF503			NM_032772.4	NCBI	2	1	Within coding region	0
Znf703	*Mus musculus*		NM_001101502.1	NCBI	2	1	Within coding region	0
Znf503			NM_145459.3	NCBI	3	2	1st Within the 5'UTR<br>2nd Within coding region	1st: no phase<br>2nd: phase 0
ZNF703	*Monodelphis domestica*		ENSMODT00000013891	Ensembl	3	2	Within coding region	1st: phase 2<br>2nd: phase 0
ZNF503			XM_001364657.3	NCBI	2	1	Within coding region	0
ZNF503	*Taeniopygia guttata*	Aves	XM_002196251.1	NCBI	2	1	Within coding region	0
znf503	*Anolis carolinensis*	Reptilia	ENSACAT00000029620	Ensembl	2	1	Within coding region	0
znf703	*Xenopus tropicalis*	Amphibia	NM_001030507.1	NCBI	2	1	Within coding region	0
znf503			NM_001079230.1	NCBI	2	1	Within coding region	0
znf503	*Danio rerio*	Actinopterygii	ENSDART00000008906	Ensembl	2	1	Within coding region	0
znf703			ENSDART00000051553	Ensembl	2	1	Within coding region	0
znf703a	*Gasterosteus aculeatus*		ENSGACT00000017593	Ensembl	2	1	Within coding region	0
znf703b			ENSGACT00000020890	Ensembl	2	1	Within coding region	0
znf503b			ENSGACT00000002922	Ensembl	2	1	Within coding region	0
znf503a			ENSGACT00000011778	Ensembl	2	1	Within coding region	0
*	*Ciona intestinalis*	Urochordata	XM_002130210.2	NCBI	2	1	Within coding region	0
*	*Branchiostoma floridae*	Cephalochordata	KU692026	GenBank	2	1	Within coding region	0
*	*Saccoglossus kowalevskii*	Hemichordata	NM_001168139.1	NCBI	2	1	Within coding region	0
*	*Strongylocentrotus purpuratus*	Echinodermata	XM_775815.3	NCBI	2	1	Within coding region	0
Elbow	*Drosophila melanogaster*	Arthropoda	FBtr0080637	Ensembl	3	2	Within coding region	1st: phase 0<br>2nd: phase 1
NocA			FBtr0080643	Ensembl	2	1	Within coding region	0
Elbow	*Tribolium castaneum*		XM_969852.1	NCBI	2	1	Within coding region	0
NocA			XM_969829.2	NCBI	2	1	Within coding region	0
*	*Lottia gigantea*	Mollusca	156395	JGI	2	1	Within coding region	0
*			156396	JGI	2	1	Within coding region	0

*			230146	JGI	2	1	Within coding region	0
*	*Capitella teleta*	Annelida	21565	JGI	2	1	Within coding region	0
*			225552	JGI	2	1	Within coding region	0
tlp-1	*Caenorhabditis elegans*	Nematoda	T23G4.1	Ensembl	4	3	Within coding region	1st: phase 0<br>2nd: phase 2<br>3rd: phase 0
*	*Nematostella vectensis*	Cnidaria	247980	JGI	2	1	Within coding region	0

* No name assigned

Supplementary Table S3 – Primers used in PCR amplifications and sequencing.

Species	Primer name	Sequence (5'- 3')	PCR product length (bp)	Touchdown PCR annealing temperatures T1/T2/T3 (°C)
Homo sapiens	ZNF703-F	ATGAGCGATTCGCCCGCTG	1773	62/60/58
	ZNF703-R	TTACTGGTATCCCAGCGCC		
	ZNF503-F	ATGAGCACAGCGCCCTC	1941	
	ZNF503-R	TCACTGATACCCCAGC		
Nematostella vectensis	Nv-F	ATGCCTACACGGACGTTCAC	1287	64/62/59
	Nv-R	TTACTTAAAGAGTCTGTGCGG		
Capitella teleta	Ct21565-F	ATCGTCTTCTCAGTACCTTCAC	1381	
	Ct21565-R	TCAGGGGGCAGCCGCTC		
	Ct225552-F	ATGCTCACCACGGCTGGATC	1445	
	Ct225552-R	CTAGGGAGTCACGGCGGC		
Lottia gigantea	Lg156395-F	ATGTTGAACGCAGCTTCGCC	1368	62/59/55
	Lg156395-R	TCACGGCACAGAACCAAGTCT		
	Lg156396-F	ATCGCAGTATCTTCACTATCCG	1462	
	Lg156396-R	TCAAGGAACAGCAGCGCCAAG		
	Lg230146-F	ATGTTGGCGAATGCTGGACC	1539	
	Lg230146-R	TCAGGGTACTGCAGCTCCTA		
Strongylocentrotus purpuratus	Sp-F	ATGCTTGCGACACGGACGC	1476	
	Sp-R	CTAGTGTGGTACTCCAGCTG		
Branchiostoma floridae	Bf-F	ATGAACAGCGTTTCTCCTGATC	1518	
	Bf-R	TCAAGGATATCCCACTGTCG		
	Bf-F2	GTCATCTGTCGCCCAGCTC	used for sequencing	---
	Bf-R2	GCATGGAGTTGGAGGGAAGG	used for sequencing	---

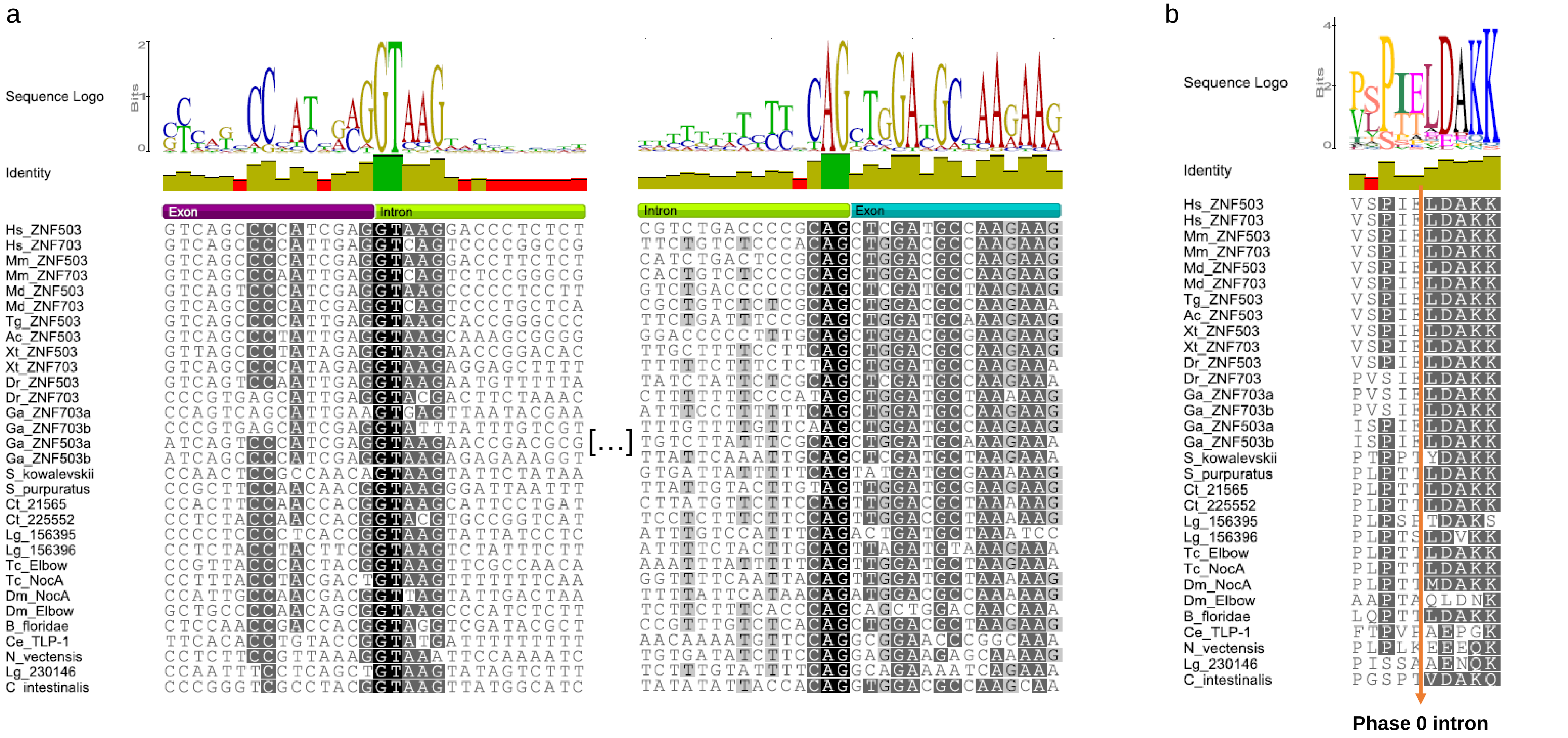

Supplementary Figure 1. Conservation of sequences around phase 0 intron on NET species. **a**) Conservation of DNA sequences near the exon–intron and intron-exon boundaries. Fifteen exon and 15 intron nucleotides of 31 representative species were aligned using the MUSCLE software. Conservation is evident at the donor and acceptor splice sites as well as the post-intron DNA sequence (right panel indicated in light blue). **b**) Conservation of protein sequences surrounding the phase 0 intron. Five pre-intron and 5 pos-intron amino acids were aligned using the MUSCLE software.

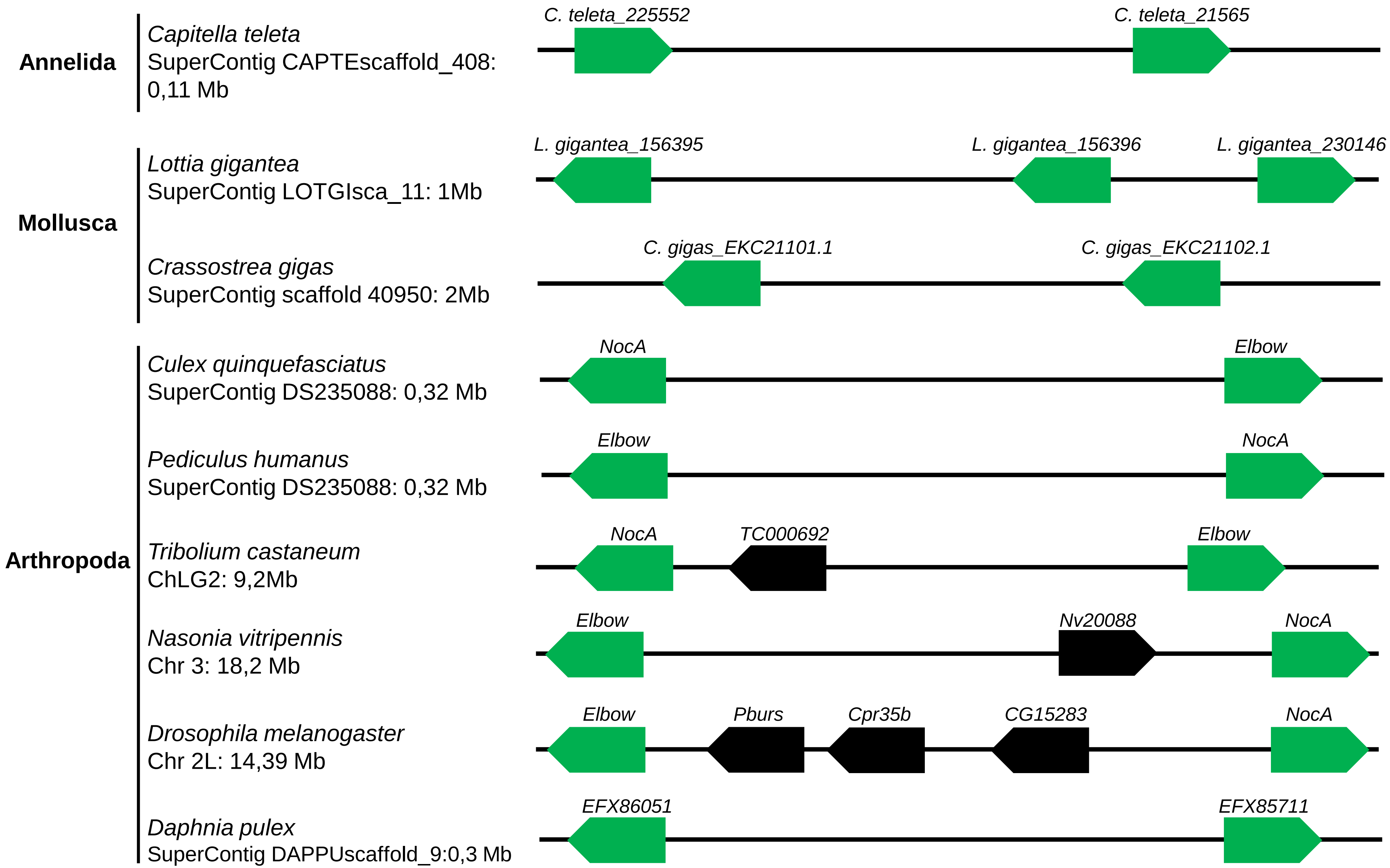

Supplementary Figure 2. Tandem duplication is a common mode of NET family gene expansion in Annelida (*C. teleta*), Mollusca (*L. gigantea* and *C. gigas*) and in some Arthropoda phylum representative species (*C. quinquefasciatus, P. humanus and D. pulex*). Green arrows indicate NET family gene orientation and order and black arrows other genes. Chromosomal (Chr) or scaffold locations are shown in megabases (Mb). Location and gene orientation were identified in the Ensembl Metazoa genome browser using the location tool together with orthologues and paralogues comparisons.
